# Aqueous Humor Cytokine Levels in Diabetic Macular Edema Patients with Cotton-Wool Spots

**DOI:** 10.1155/2019/8137417

**Published:** 2019-12-21

**Authors:** Young-Gun Park, Donghyun Jee, Jin-woo Kwon

**Affiliations:** ^1^Department of Ophthalmology, Seoul St. Mary's Hospital, College of Medicine, Catholic University of Korea, Seoul, Republic of Korea; ^2^Department of Ophthalmology, St. Vincent's Hospital, College of Medicine, Catholic University of Korea, Seoul, Republic of Korea

## Abstract

**Purpose:**

To determine the characteristics of diabetic macular edema (DME) patients with cotton-wool spots (CWS).

**Methods:**

We classified 80 treatment-naïve DME patients according to whether or not they had CWS involving macula and then compared the concentrations of interleukin- (IL-) 1*β*, IL-6, IL-8, IL-10, IL-17, placental growth factor, vascular endothelial growth factor (VEGF), and intercellular adhesion molecule (ICAM)-1 in the aqueous humor between the groups, as well as optical coherence tomography (OCT) findings, baseline characteristics, and intravitreal bevacizumab responsiveness.

**Results:**

Aqueous levels of ICAM-1 and VEGF in the group with CWS were significantly higher than those in the non-CWS (control) group (*p* < 0.001 and *p* = 0.006, respectively). In multiple logistic regression analysis to identify factors associated with CWS, the aqueous ICAM-1 (≥0.36 ng/mL) was significantly associated with CWS (odds ratio = 13.26, *p* < 0.001). Based on OCT, ellipsoid zone (EZ) disruption distribution was significantly different between the two groups (*p* = 0.038). Regarding responsiveness to treatment, although there was no significant difference in central subfield thickness between the two groups after treatments, the best-corrected visual acuity was worse in the group with CWS.

**Conclusions:**

The presence of CWS was accompanied by higher levels of aqueous ICAM-1. Based on OCT, EZ disruption was greater in DME patients with CWS, and their short-term visual prognosis was poorer.

## 1. Introduction

Diabetic macular edema (DME) is the major cause of vision impairment in diabetic patients [1, 2]. Alteration of the blood-retina barrier is a hallmark of DME, which is characterized by loss of pericytes and breakdown of the endothelial junction [3]. Multiple cells, cytokines, and growth factors are involved in the pathogenesis of DME, which affects the neurovascular system [4].

Cotton-wool spots (CWS), also known as soft exudate, are accumulations of axoplasmic debris related to microinfarcts induced by arteriolar occlusion [5]. Although CWS are the common findings in the nonproliferative stage of diabetic retinopathy (DR), they could be associated with disease progression [6]. Many studies have shown that the aqueous humor of DME patients contains elevated levels of inflammatory cytokines, growth factors, and matrix metalloproteinases, which are associated with the status of the retina [7–9]. The individual roles played by these factors in the pathogenesis of DME remain unclear, although many studies have sought to determine their mechanisms of action in detail.

We thus determined the levels of IL-1*β*, IL-6, IL-8, IL-10, IL-17, placental growth factor (PlGF), vascular endothelial growth factor (VEGF), and intercellular adhesion molecule (ICAM)-1 in the aqueous humor of 21 DME patients with CWS and compared them with those of 59 DME patients without CWS.

## 2. Methods

The study protocol adhered to the tenets of the Declaration of Helsinki. The protocol was approved by the Institutional Review/Ethics Board of the Catholic University of Korea. All the participants gave written informed consent for the use of their clinical records.

We enrolled patients with treatment-naïve center-involving DME (ciDME) eyes and excluded the eyes with macular edema due to other causes or any history of uveitis or intraocular surgery. We classified them according to whether or not they had CWS involving macula.

**Figure 1:**
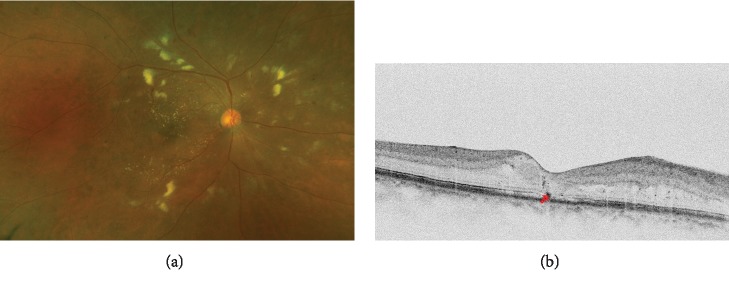
A representative patient who had diabetic macular edema with cotton-wool spots (CWS). (a) The baseline fundus photo image shows multiple CWS around fovea. (b) The baseline spectral domain-optical coherence tomography (SD-OCT) image shows focal ellipsoid zone disruption (red arrow).

We measured glycated hemoglobin levels, and all the patients received ophthalmic examinations, which included the measurement of the best-corrected visual acuity (BCVA) and a fundus examination. The central subfield thickness (CST) was measured using optical coherence tomography (OCT; Cirrus High-Definition OCT; Carl Zeiss Meditec, Dublin, CA, USA). The hyperreflective foci (HF) were manually measured within 1,500 *μ*m, and ellipsoid zone (EZ) disruption was manually checked within 1,000 *μ*m using a horizontal scan centered on the fovea (Figure 1) [10].

### 2.1. Assay of Cytokines and Growth Factors

We measured the concentrations of interleukin- (IL-) 1*β*, IL-6, IL-8, IL-10, IL-17, PlGF, VEGF, and ICAM-1 in 75 *μ*L of the aqueous humor. The antibodies were immobilized on beads, and the samples with 75 *μ*L Calibrator Diluent RD6-52 (R&D Systems, Minneapolis, MN, USA) were added to the preparations. The samples were then incubated for 2 h after adding the beads and incubated for 1 h after adding the detection antibodies; then, they were incubated for 30 min after adding the streptavidin-phycoerythrin reagent. The samples were read using the Luminex xMAP system (Luminex, Austin, TX, USA) [7].

### 2.2. Statistical Evaluation

Statistical analyses were performed using SPSS statistical software for Windows, version 21.0 (SPSS, Chicago, IL, USA). The *t*-test, Mann–Whitney *U* test, and chi-square test were used to compare the values or the ratios of the patient subgroups. The signed-rank test was used to compare changes in CST and BCVA. Logistic regression was employed to identify factors associated with CWS.

## 3. Results

We enrolled 80 treatment-naïve (ciDME) eyes of 80 patients. The mean age was 57.54 ± 10.21 years, and there were 39 males and 41 females. In the DR staging, 45 patients had proliferative DR (56.25%) and 35 had nonproliferative DR (43.75%). The mean BCVA (logMAR) was 0.64 ± 0.35, and the mean CST was 408.74 ± 122.94 *μ*m at baseline. When classifying the DME morphology, 33 patients had cystoid macular edema and 47 had diffuse retinal thickening.

There were 21 eyes with CWS involving macula among the DME patients and 59 eyes without CWS. There was no significant difference in age, sex, DR stage distribution, glycated hemoglobin level, BCVA, or CST between the two groups. Although there was no significant difference in CST after three consecutive intravitreal bevacizumab (IVB) injections between the two groups (*p* = 0.861), the BCVA (logMAR) of the CWS group was significantly worse than that of the group without CWS (*p* = 0.006). The systemic and ocular characteristics of the patients in each group are summarized in Table 1.

**Figure 2:**
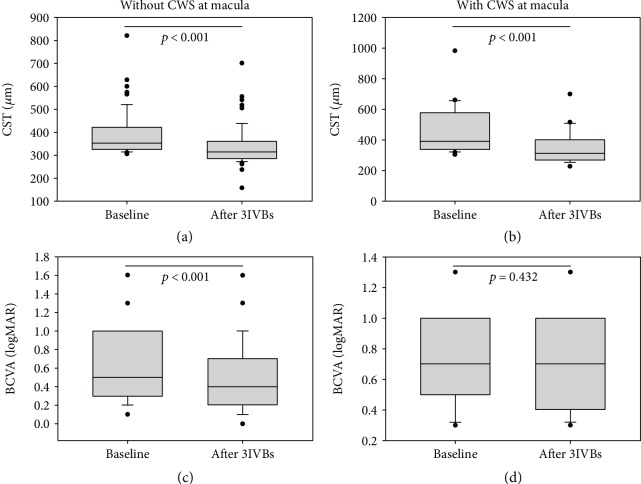
Box and whisker plots for central subfield thickness (CST) and changes in best-corrected visual acuity (BCVA) in patients with diabetic macular edema, with or without cotton-wool spots (CWS), after three consecutive monthly intravitreal bevacizumab (IVB) injections. The circles represent outliers. (a, b) The CST was significantly reduced after IVB injections in both groups (all, *p* < 0.001). (c, d) The BCVA improved significantly after IVB injection in the group without CWS, but did not significantly change in the group with CWS (*p* < 0.001 and *p* = 0.432, respectively).

The CST reduction after three consecutive monthly IVBs of the group with CWS was ‐97.38 ± 129.59 *μ*m, and that of the group without CWS was ‐49.00 ± 79.56 *μ*m. The average CST reduction did not significantly differ between the two groups (*p* = 0.136). The CST values were significantly different, before versus after treatment, in each group (both, *p* < 0.001) (Figure 2).

The BCVA (logMAR) improved by as much as ‐0.033 ± 0.143 in the group with CWS and by ‐0.110 ± 0.149 in the group without CWS (control). There was a statistically significant difference (*p* = 0.044) in the degree of change in the BCVA between the two groups. The change was significant in the control group (*p* < 0.001), but not in the group with CWS (*p* = 0.432) (Figure 2).

### 3.1. SD-OCT Morphological Findings

The number of HF patients was not significantly different between the groups, based on OCT (*p* = 0.570), and nor was the distribution of DME types (*p* = 1.000). However, the proportion of EZ disruption was significantly different between the two groups (*p* = 0.038) (Table 1).

### 3.2. Aqueous Concentration of Cytokines

When comparing the cytokine levels in the aqueous humor, the median levels of ICAM-1, IL-1*β*, IL-6, and VEGF were higher in the group with versus without CWS. The median levels of ICAM-1 and VEGF in the group with CWS were significantly higher than those in the control group (*p* < 0.001 and *p* = 0.006, respectively). However, the levels of other cytokines (IL-1*β*, IL-6, IL-8, IL-10, IL-17, and PlGF) did not differ between the two groups (Table 1). When using multiple logistic regression analyses to identify factors associated with CWS, the aqueous ICAM-1 (≥0.36 ng/mL) was significantly associated with CWS (odds ratio = 13.26, *p* < 0.001) (Table 2).

## 4. Discussion

CWS are the common fundus finding of DR patients and are thought to be a representative finding of moderate nonproliferative DR [11]. It is well-known that CWS are associated with progression of DR, but the mechanism remains unknown [11]. In the present study, we showed that the aqueous humor of DME patients with CWS showed higher levels of VEGF and ICAM-1 than that of the control group. Because VEGF levels are associated with endothelial damage of the blood-retina barrier, as well as neovascularization, the higher aqueous levels of VEGF in the CWS group could be involved in the mechanism of progression.

In our study, although there was no significant group difference in the average CST and BCVA values, the group with CWS had a worse BCVA and higher CST at baseline than the control group. The CST reduction after anti-VEGF treatment was nearly twofold greater in the group with CWS compared with the control group (97.38 ± 129.59 *μ*m vs. 49.00 ± 79.56 *μ*m, respectively). Despite the greater CST reduction, the group without CWS showed significant improvement of the BCVA (*p* < 0.001), but no significant change of the BCVA in CWS group, after versus before anti-VEGF treatment (*p* = 0.432). We suggest that the lower BCVA at baseline and lack of improvement of the BCVA after treatment could be due to the EZ disruption. The EZ is an indicator of photoreceptor layer health, and disruption therein is reported to be associated with visual prognosis in DME patients [10]. Recent studies have shown that disorganization of retinal inner layers could be associated with ischemia [12]. Although CWS could be a manifestation of microinfarcts in the superficial retina, CWS and EZ disruption commonly represent the manifestations of retinal ischemia. In the present study, the EZ disruption was associated with the presence of CWS, so the CWS might also be associated with the visual prognosis.

The aqueous ICAM-1 and VEGF levels were elevated in the group with CWS, and multivariate logistic regression revealed an association between ICAM-1 levels and CWS. ICAM-1, one of the inflammatory markers, promotes the infiltration of leukocytes into inflammatory tissues [13]. In diabetes, the blood levels of ICAM-1 are increased, which could be a predictor of macrovascular complications such as myocardial infarct or stroke [14, 15]. In terms of DR, ICAM-1 is known to potentiate retinal vascular leukocyte adhesion, increase vascular permeability, upregulate VEGF, and promote breakdown of blood-retina barrier during the pathogenesis of DME [16, 17]. Previous studies reported that increased aqueous humor ICAM-1 and VEGF levels correlated with the severity of DME or DR [7, 9, 18]. Another study showed that an increase in blood VEGF and ICAM-1 levels is associated with a DR severity and EZ disruption [17]. Furthermore, recent studies reported that aqueous ICAM-1 and VEGF levels could be biomarkers to predict the anatomic response to anti-VEGF agents in DME [19, 20]. Based on these investigations, VEGF and ICAM-1 might play a key role in the progression of DR.

Although vitreous samples could provide a more definitive retinal pathology, studies using vitreous are few because they need vitrectomies [21–23]. Studies using vitreous samples also showed that the levels of inflammatory cytokines and growth factors increased in correlation with DME severity and increased VEGF level could be a predictable factor for progression of DR [22, 23]. Additionally, there was a study that showed significant relationships in cytokine levels between aqueous humor and vitreous sample [24].

There were a few limitations to this study. First, the release of a particular cytokine could be a result of the disease process, rather than a causal factor in the disease. The role of cytokines in DME pathogenesis cannot be definitively proven by their detection in aqueous samples. Second, our sample size was small. Increasing the sample size would have increased the statistical power, allowing the relationships of the variables studied to be determined more definitively.

In conclusion, our study showed that the presence of CWS is accompanied by increased levels of aqueous VEGF and ICAM-1. In addition, EZ disruption was greater in DME patients with CWS than in controls, and the short-term visual prognosis was poor despite an acceptable reduction of CST. Further studies including larger samples and a greater number of cytokines should be conducted to confirm the results of the present study.

## Figures and Tables

**Table 1:** Demographics and clinical characteristics of DME patients classified with retinal morphology.

			DME without CWS (*n* = 59)	DME with CWS (*n* = 21)	*p*
Systemic factors	Sex (male : female)		32 : 27	9 : 12	0.521
	Age (years)		57.0 [52.5; 63.5]	61.0 [55.0; 64.0]	0.599
	HbA1C (%)		7.79 ± 1.13	7.21 ± 1.24	0.054
	Hypertension		23 (38.98%)	13 (61.91%)	0.119

OCT findings	Number of HF		5.0 [3.0; 10.0]	5.0 [4.0; 10.0]	0.570
	DME type (DRT : CME)		35 : 24	12 : 9	1.000
	EZ disruption grade	(-)	39 (66.10%)	7 (33.33%)	0.038
		(+)	20 (33.90%)	14 (66.67%)	

Aqueous humor cytokines and growth factors	ICAM-1 (ng/mL)		0.00 [0.00; 0.00]	0.36 [0.00; 2.87]	<0.001
	IL-1*β* (pg/mL)		0.00 [0.00; 0.43]	0.17 [0.00; 0.17]	0.591
	IL-6 (pg/mL)		7.96 [4.16; 18.64]	16.32 [4.98; 31.88]	0.281
	IL-8 (pg/mL)		13.65 [9.77; 23.84]	11.37 [8.16; 24.43]	0.364
	IL-10 (pg/mL)		0.65 [0.19; 1.32]	0.53 [0.26; 0.93]	0.700
	IL-17 (pg/mL)		1.36 [0.54; 1.96]	0.54 [0.00; 2.55]	0.356
	PlGF (pg/mL)		2.85 [1.92; 3.97]	2.85 [2.32; 7.70]	0.477
	VEGF (pg/mL)		48.18 [22.62; 80.33]	92.73 [59.70; 184.46]	0.006

Ocular factors	Baseline BCVA (logMAR)		0.50 [0.30; 1.00]	0.70 [0.50; 1.00]	0.053
	BCVA after IVBs (logMAR)		0.40 [0.20; 0.70]	0.70 [0.40; 1.00]	0.006
	Baseline CST (*μ*m)		353.0 [326.5; 419.5]	391.0 [347.0; 574.0]	0.054
	CST after IVBs (*μ*m)		314.0 [286.5; 358.5]	313.0 [272.0; 398.0]	0.861
	DMR (NPDR : PDR)		26 : 33	9 : 12	1.000

Values are expressed as mean ± standard deviation or median and interquartile range, as appropriate. DME: diabetic macular edema; CWS: cotton-wool spot; HbA1c: glycated hemoglobin; HF: hyperreflective foci; CME: cystoid macular edema; DRT: diffuse retinal thickening; EZ: ellipsoid zone; ICAM: intercellular adhesion molecule; IL: interleukin; PlGF: placental growth factor; VEGF: vascular endothelial growth factor; BCVA: best-corrected visual acuity; IVB: intravitreal bevacizumab; CST: central subfield thickness; DMR: DM retinopathy; NPDR: nonproliferative diabetic retinopathy; PDR: proliferative diabetic retinopathy.

**Table 2:** Results of logistic regression of the effects of CWS in DME patients.

	Category	*n* (%)	Univariate		Multivariate	
			OR (95% CI)	*p*	OR (95% CI)	*p*
Sex	Female	41 (51.25%)	Reference			
	Male	39 (48.75%)	1.58 (0.58, 4.42)	0.372		

Age (years)	<60	47 (58.75%)	Reference			
	≥60	33 (41.25%)	1.85 (0.68, 5.14)	0.231		

HbA1c	≤7	23 (28.75%)	Reference			
	>7	57 (71.25%)	0.41 (0.14, 1.20)	0.101		

DMR stage	NPDR	35 (43.75%)	Reference			
	PDR	45 (56.25%)	1.05 (0.39, 2.93)	0.924		

Hypertension	(+)	36 (45.00%)	Reference		Reference	
	(-)	44 (55.00%)	2.54 (0.93, 7.34)	0.074	2.34 (0.70, 8.31)	0.171

ICAM-1 (ng/mL)	<0.36	65 (81.25%)	Reference		Reference	
	≥0.36	15 (18.75%)	15.12 (4.29, 64.09)	<0.001	13.26 (3.58, 59.16)	<0.001

IL-1*β* (pg/mL)	<0.17	46 (57.50%)	Reference			
	≥0.17	34 (42.50%)	1.72 (0.63, 4.77)	0.289		

IL-6 (pg/mL)	<8.40	40 (50.00%)				
	≥8.40	40 (50.00%)	1.48 (0.54, 4.12)	0.447		

IL-8 (pg/mL)	<13.14	40 (50.00%)	Reference			
	≥13.14	40 (50.00%)	0.68 (0.24, 1.84)	0.447		

IL-10 (pg/mL)	<0.65	36 (45.00%)	Reference			
	≥0.65	44 (55.00%)	0.67 (0.24, 1.82)	0.430		

IL-17 (pg/mL)	<1.36	36 (45.00%)	Reference			
	≥1.36	44 (55.00%)	0.67 (0.24, 1.82)	0.430		

VEGF (pg/mL)	<59.65	40 (50.00%)	Reference		Reference	
	≥59.65	40 (50.00%)	3.40 (1.20, 10.69)	0.026	0.321 (0.95, 12.41)	0.071

PlGF (pg/mL)	<2.85	36 (45.00%)	Reference			
	≥2.85	44 (55.00%)	1.47 (0.54, 4.20)	0.460		

CWS: cotton-wool spot; DME: diabetic macular edema; OR: odds ratio; CI: confidence interval; ICAM: intercellular adhesion molecule; IL: interleukin; VEGF: vascular endothelial growth factor; PlGF: placental growth factor.

## Data Availability

The data used to support the findings of this study are available from the corresponding author upon request.
